# Supplementary material for: Monitoring the opioid epidemic via social media discussions
Source: NPJ Digit Med. 2025 May 15;8:284. doi: 10.1038/s41746-025-01642-x (PMC12081907; doi:10.1038/s41746-025-01642-x)
Supplement: Supplementary file 1 — NPJ_supplement [file 41746_2025_1642_MOESM1_ESM.pdf]

Supplementary Figures

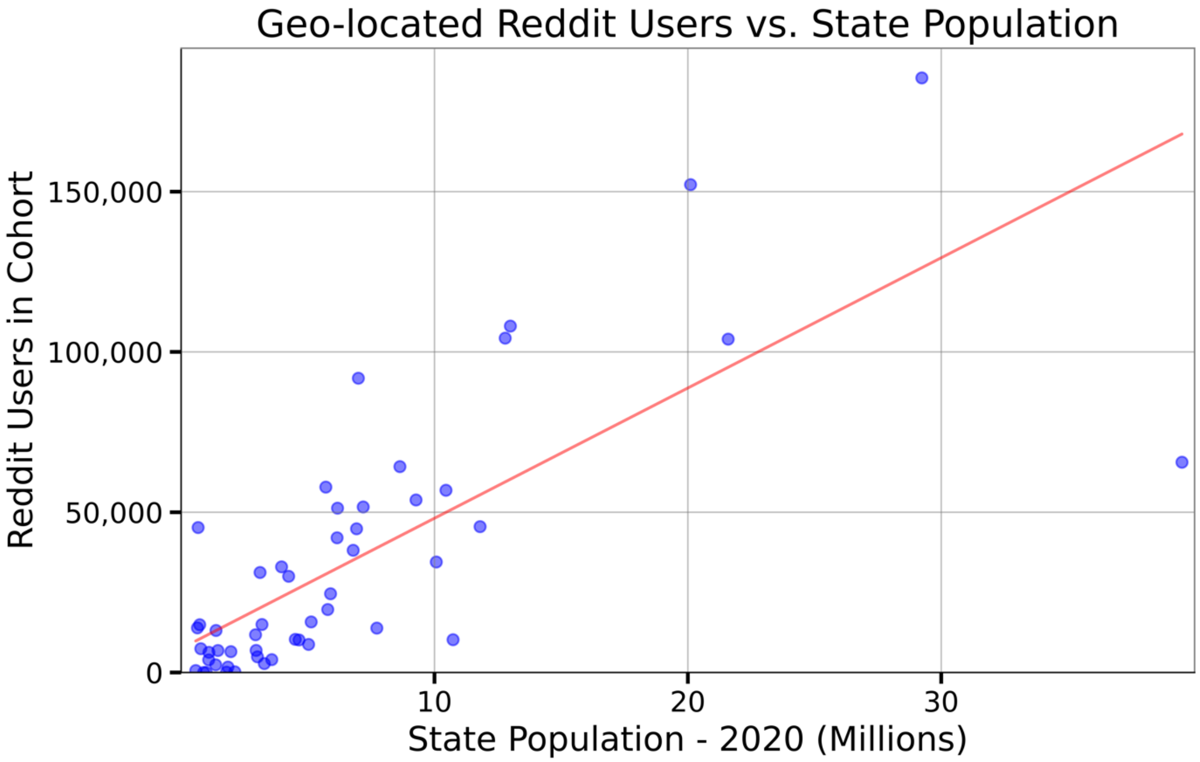

Supplementary Figure 1: State populations in 2020 (x-axis) versus the number of users assigned to that state in our cohort of geolocated users (Kendall's Tau = 0.595). Four states had no observed users and were added to the plot with zero users.

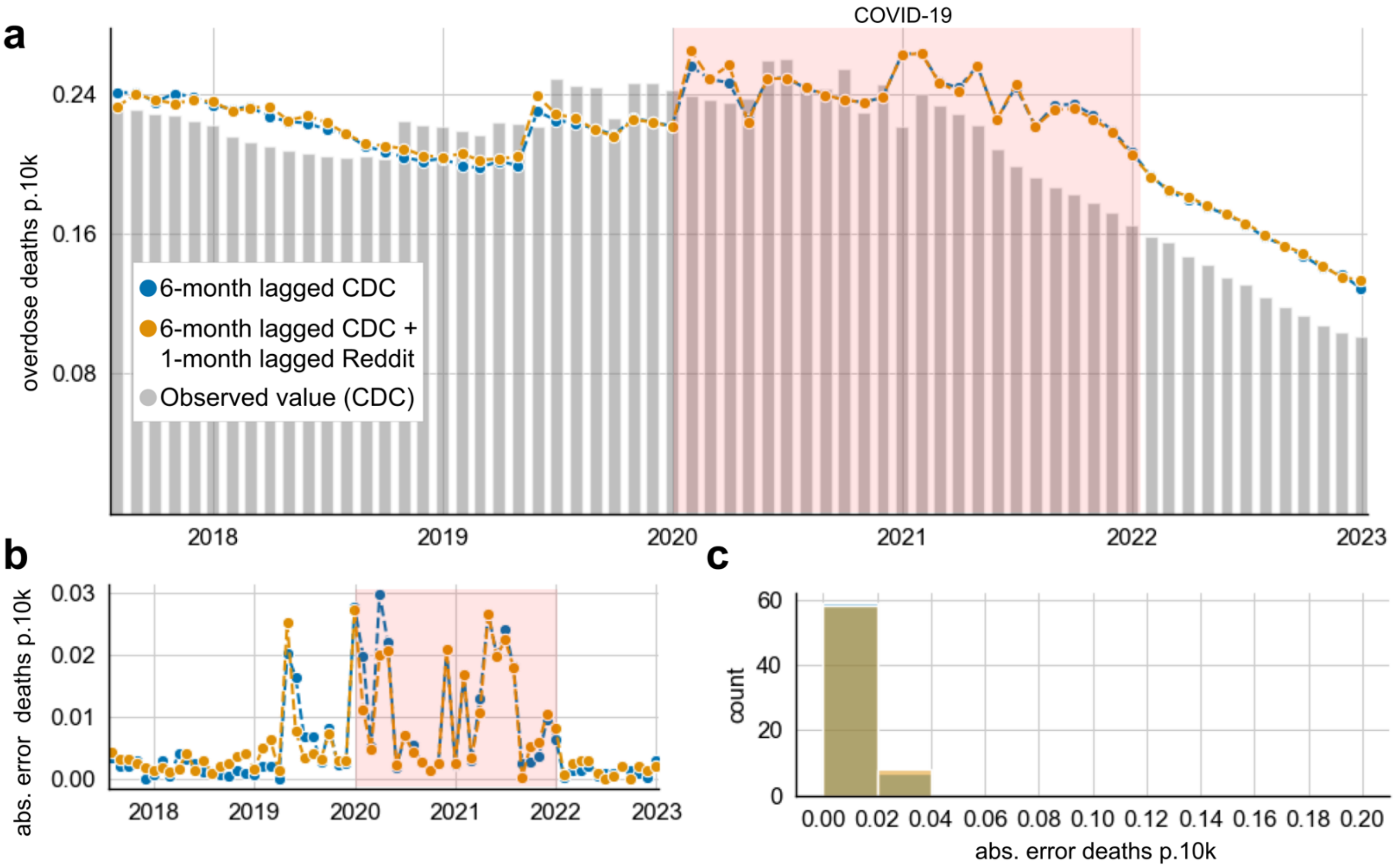

Supplementary Figure 2. Autoregressive Integrated Moving Average (ARIMA) simulations for heroin. a) Predicted monthly overdose death rates per 10,000 people of rolling-origin forecast models are shown based on 1-month prediction horizons. Observed mortality is shown in grey, and monthly overdose death rates per 10,000 people as predicted by ARIMA models fitted on 6-month-lagged CDC overdose death (blue) are shown along with predictions from a model that additionally included 1-month-lagged Reddit data (orange). **b**) shows the absolute errors over time of the monthly overdose deaths per 10,000 people predicted by the lagged CDC model alone (in blue) and the combined CDC/Reddit model (in orange); **c**) shows the corresponding distributions. The combination Reddit/CDC model showed improved predictive accuracy for overdose death rates compared to the lagged CDC data alone, which did not rise to statistical significance ($p = 0.200$). The average absolute error for CDC alone was 0.0063, and 0.0062 for the combination model (in monthly overdose death rates per 10,000 people). In both models, we see notably decreased performance during the COVID-19 years from 2020 until 2022. Before 2020, the combination model outperformed the CDC-alone model (not significant at $p = 0.105$), with average absolute errors of 0.0048 and 0.0043 monthly normalized overdose death rates for CDC alone and CDC/Reddit, respectively.

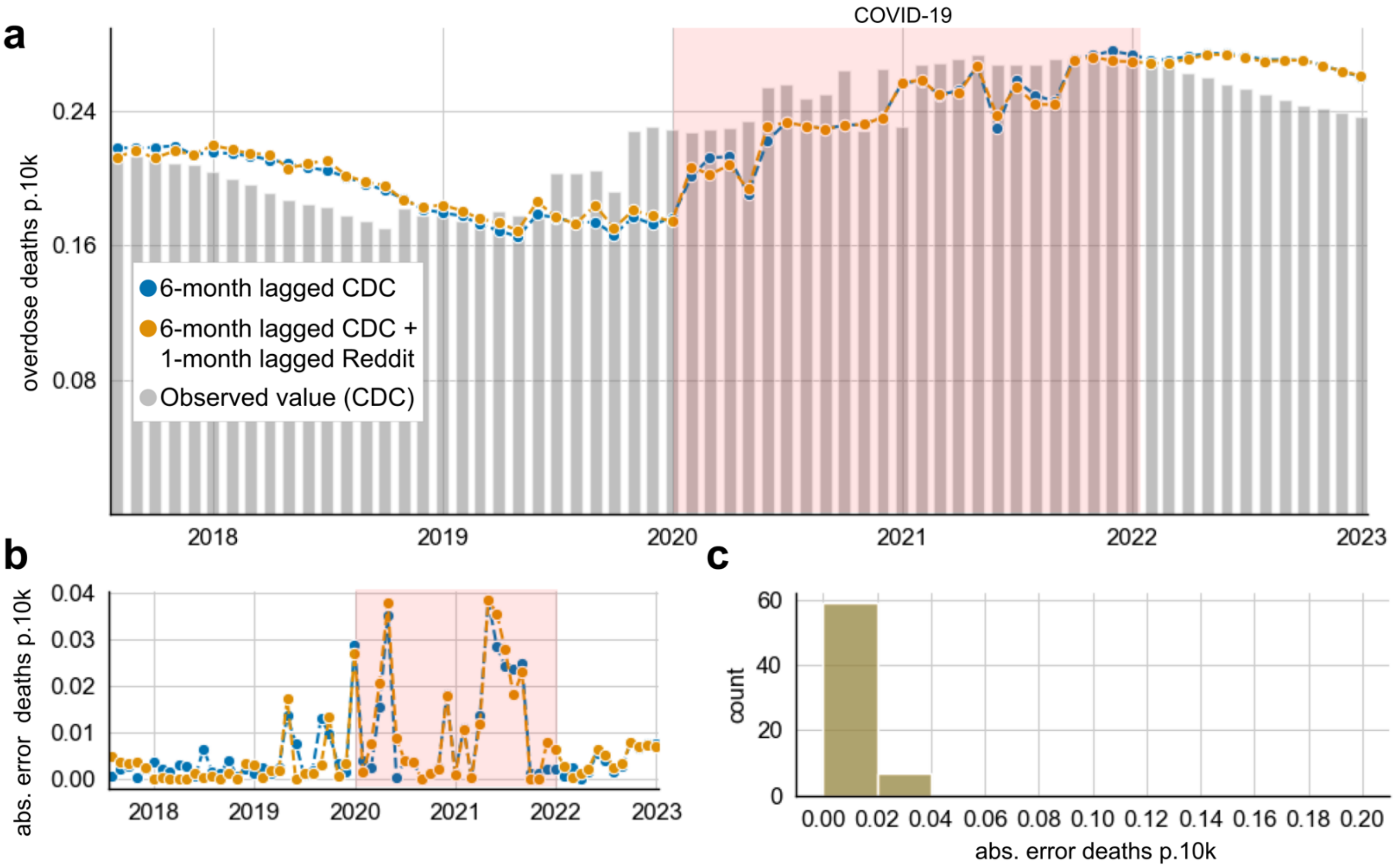

Supplementary Figure 3. Autoregressive Integrated Moving Average (ARIMA) simulations for natural and semi-synthetic opioids. **a**) Predicted monthly overdose death rates per 10,000 people of rolling-origin forecast models are shown based on 1-month prediction horizons. Observed mortality is shown in grey, and monthly overdose death rates per 10,000 people as predicted by ARIMA models fitted on 6-month-lagged CDC overdose death (blue) are shown along with predictions from a model that additionally included 1-month-lagged Reddit data (orange). **b**) shows the absolute errors over time of the monthly overdose deaths per 10,000 people predicted by the lagged CDC model alone (in blue) and the combined CDC/Reddit model (in orange); **c**) shows the corresponding distributions. The distribution of absolute errors of the monthly overdose deaths per 10,000 people of the lagged CDC model alone (in blue) and the combined CDC/Reddit model (in orange). The Reddit/CDC model did not show improved accuracy over the CDC-only model in predicting overdose death rates ($p = 0.865$, Supplemental Figure 3). In both models, we see notably decreased performance during the COVID-19 years from 2020 until 2022. Before 2020, the combination model outperformed the CDC-alone model (not significant at $p = 0.221$), with average absolute errors of 0.00344 and 0.00341 for CDC alone and CDC/Reddit, respectively.
